# Frontal Cortical Modulation of Temporal Visual Cross-Modal Re-organization in Adults with Hearing Loss

**DOI:** 10.3390/brainsci10080498

**Published:** 2020-07-30

**Authors:** Julia Campbell, Anu Sharma

**Affiliations:** 1 Central Sensory Processes Laboratory, Department of Communication Sciences and Disorders, University of Texas at Austin, 2504 Whitis Ave a1100, Austin, TX 78712, USA; julia.campbell@austin.utexas.edu; 2 Anu Sharma, Brain and Behavior Laboratory, Institute of Cognitive Science, Department of Speech, Language and Hearing Science, University of Colorado at Boulder, 409 UCB, 2501 Kittredge Loop Drive, Boulder, CO 80309, USA

**Keywords:** sLORETA, high-density EEG, mild-moderate hearing loss, visual evoked potentials, speech perception, visual cross-modal re-organization, top-down modulation, frontal cortex, cognitive load, listening effort

## Abstract

Recent research has demonstrated frontal cortical involvement to co-occur with visual re-organization, suggestive of top-down modulation of cross-modal mechanisms. However, it is unclear whether top-down modulation of visual re-organization takes place in mild hearing loss, or is dependent upon greater degrees of hearing loss severity. Thus, the purpose of this study was to determine if frontal top-down modulation of visual cross-modal re-organization increased across hearing loss severity. We recorded visual evoked potentials (VEPs) in response to apparent motion stimuli in 17 adults with mild-moderate hearing loss using 128-channel high-density electroencephalography (EEG). Current density reconstructions (CDRs) were generated using sLORETA to visualize VEP generators in both groups. VEP latency and amplitude in frontal regions of interest (ROIs) were compared between groups and correlated with auditory behavioral measures. Activation of frontal networks in response to visual stimulation increased across mild to moderate hearing loss, with simultaneous activation of the temporal cortex. In addition, group differences in VEP latency and amplitude correlated with auditory behavioral measures. Overall, these findings support the hypothesis that frontal top-down modulation of visual cross-modal re-organization is dependent upon hearing loss severity.

## 1. Introduction

Auditory deprivation, or hearing loss, has been shown to affect adults in several ways, including increasing risk for social isolation, listening effort and fatigue, cognitive decline, and cross-modal cortical re-organization [1,2,3,4,5]. The latter of these, cross-modal cortical re-organization, occurs when one intact sensory modality (e.g., vision) recruits and re-purposes cortical regions of a different sensory modality (e.g., audition), due to a period of deprivation or lack of stimulation in that modality [6]. In hearing loss, the recruitment of auditory cortical regions by visual processes has been well-documented in both animals and humans [7,8,9,10,11], and has further been related to performance enhancement in various visual tasks [12,13,14,15,16]. This heightened visual function is thought to be compensatory in nature, likely to subserve communication purposes, such as auditory-visual integration, when auditory input is significantly decreased [14,15,16,17]. Visual recruitment of the central auditory system was first demonstrated in congenitally deaf adults, who showed activation of the auditory cortex in response to visual motion [7,8,9,18]. This cortical response was described to occur solely because of auditory deprivation, or a bottom-up sensory mechanism, rather than as a byproduct of a manual communication modality (i.e., American Sign Language) [12]. Thus, auditory deprivation has been indicated to drive visual recruitment of auditory cortex.

Later, as cochlear implantation became a successful option for the restoration of auditory input via electrical stimulation of the VIII nerve, implanted adults with acquired profound hearing loss were found to also demonstrate evidence of visual re-organization, with visual input processed in both the tvisual and auditory cortices [19,20,21,22,23,24]. This outcome, similar to that reported in congenitally deaf adults, indicates that auditory deprivation does not have to be present from birth to induce visual recruitment of auditory cortex, but that recruitment may also take place in response to acquired, shorter durations of deprivation. Furthermore, several studies have described a significant relationship between the amount of visual recruitment of auditory cortex and speech perception performance. In other words, cross-modal reorganization of the auditory cortex by vision is correlated with lower speech perception in cochlear-implanted adults [19,21,22,23,25,26]. This finding may be due to a competition for cortical resources, in that, insufficient auditory cortical mechanisms are preserved to process incoming auditory input [22,27].

While visual cross-modal re-organization clearly occurs in profound hearing loss, several studies have reported this type of plasticity to be intiated in the early stages of auditory deprivation, or mild-moderate hearing loss [2,15,28,29]. These studies revealed that visual re-organization of the auditory cortex seems to occur quite early in sensory deprivation, with as little as mild deprivation. Furthermore, similar to cochlear-implanted adults, visual recruitment of auditory cortex in mild-moderate hearing loss is also associated with difficulties in auditory-only speech perception and listening effort, indicating that cortical re-organization may be an important clinical factor in auditory outcomes for clinical patients without profound auditory deprivation [2,14,17,28,29,30].

In addition to visual recruitment of auditory networks in mild-moderate hearing loss, recent research has identified frontal and pre-frontal cortical involvement in the processing of visual information for this population [28,31], raising the question of whether top-down modulatory mechanisms may be related to cross-modal re-organization. Using fMRI, Rosemann and Thiel (2018) found increased frontal activation of cognitive networks in response to visual sentences for adults with mild-moderate hearing loss which was correlated with both the degree of loss and listening effort. Similarly, Glick and Sharma (2020) showed frontal and pre-frontal sources to underlie cortical visual evoked potentials (VEPs) elicited passively by apparent motion in mild-moderate hearing loss. Right temporal cortex was also responsive to the visual pattern in the hearing loss participants, consistent with visual cross-modal re-organization. The authors therefore hypothesized that frontal and pre-frontal networks may act as a top-down mechanism to modulate visual processing networks that become active in auditory deprivation due to stimulus relevance (i.e., the salience of visual information versus auditory information). Indeed, in typical populations, similar cognitive networks are indicated in the early modulation of higher-order visual tasks such as visual spatial attention and working memory [32], word reading [33], and scene analysis [34].

Taken together, it appears that both bottom-up sensory mechanisms (i.e., visual recruitment of auditory systems driven by auditory deprivation) and top-down modulatory mechanisms (i.e., visual recruitment of cognitive networks) are involved in the processing of visual information in hearing loss. However, the role of the degree of auditory deprivation in this interaction remains unknown. For example, it is unclear whether top-down cognitive mechanisms are active in visual cross-modal re-organization in mild hearing loss, or whether moderate auditory deprivation is required for initation of this compensatory mechanism. Given that age-related hearing loss is typically insidious and long-standing before it becomes a clinical problem, a better understanding of the underlying trajectory of compensatory networks will provide clinically relevant information. Therefore, in the current study, we aimed to observe frontal and pre-frontal top-down resources activated by visual input as a function of mild versus moderate hearing loss and speech perception outcomes. We recorded visual evoked potentials (VEPs) via high-density electroencephalography EEG in response to apparent motion in a group of adults with mild and moderate hearing loss. Source localization analyses were conducted to view activated cortical networks between frontal and temporal regions in the two groups, and VEP responses in frontal regions of interest (ROIs) were created to assess frontal cortical responses. VEP responses were correlated with auditory behavioral measures, including speech perception in noise and pure-tone thresholds. We hypothesized that as hearing loss and speech perception worsened, frontal cortical regions, in addition to auditory cortical areas in the temporal cortex, would be increasingly activated to assist in processing of visual sensory information.

## 2. Materials and Methods

### 2.1. Participants and Ethics Statement

Seventeen adults in the age range of 38 to 78 years participated in this study, which was approved by the University of Colorado at Boulder Institutional Review Board. The research was conducted in accordance with the Human Subjects Institutional Review Board (IRB) protocol 0906.16, approved 25 June 2010, at the University of Colorado at Boulder. All participants provided written consent. Each participant received a clinical audiometric evaluation, followed by a speech perception-in-noise assessment and EEG measurements.

Of the seventeen participants, ten participants (mean age and standard deviation: 58.6 +/− 9.5 years; range: 38.4–69.6 years) presented with a clinically mild, sensorineural age-related hearing loss bilaterally. On average, pure tone thresholds for this group were below 25 dB Hearing Level (HL) for frequencies 500–2000 Hz, denoting normal hearing in this range [2,35,36]. For the higher frequencies of 4000 and 8000 Hz, pure tone thresholds increased to the range of 25–36 dB HL, or a mild hearing loss (MILD) [36]. A second group of seven participants (mean age and standard deviation: 66 +/− 7 years; range: 54.5–78 years) presented with a clinically moderate sensorineural hearing loss bilaterally. These participants also demonstrated average pure tone thresholds under 25 dB HL for frequencies 500–2000 Hz, indicating normal hearing in this range. For the frequency range of 4000–8000 Hz, pure tone thresholds increased to levels of 45–52 dB HL, or a moderate hearing loss (MOD) [36]. At the time of testing, participants did not report receiving clinical services for their hearing loss. Those who were diagnosed with hearing loss through the study received counseling from a state-licensed clinical audiologist (first author) and referrals to audiology clinics for consideration of amplification.

**Figure 1:**
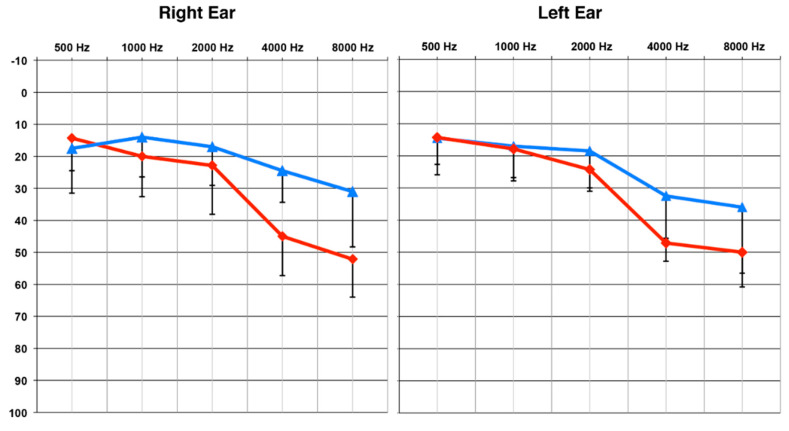
Group Mean Pure Tone Thresholds. The MILD hearing loss group (n = 10) is designated by the blue line and the MOD hearing loss group (n =7) by the red line. Standard deviations are represented by negative-going error bars at each frequency. Tested frequencies (Hz) are shown on the horizontal axis and intensity levels (dB HL) on the vertical axis.

Average audiograms for the two groups are shown in Figure 1. Pure tone averages (PTA) at 4000 and 8000 Hz, in the best ear, were compared between groups using a Mann–Whitney U test [37], due to the unequal sample sizes. The MOD group was found to have a significantly worse (or higher) threshold than the MILD group (*U* = 70, *Z* = 3.42, *p* < 0.001). There was no significant difference in age between the participants in the MILD and MOD groups (*U* = 49, *Z* = 1.37, *p* > 0.05), and no impairments in visual acuity or neurological function were reported.

### 2.2. Auditory Behavioral Testing: Measure of Speech Perception in Noise

The QuickSIN™ measure [38], which is a clinical assessment of auditory acuity in background noise for adults, was utilized to quantify each participant’s speech perception performance in background noise. Participants were instructed to face a speaker at 0° azimuth and repeat two recorded sentence lists (six sentences each) presented at 65 dB HL. For each sentence presentation, background multitalker babble was varied to determine the signal-to-noise ratio (SNR) required by the participant to accurately repeat 50% of the sentences. The SNR values began at 25 dB and decreased in 5 dB increments to 0 dB. The SNR threshold from the two lists was calculated and averaged for each participant. Overall, the lower the SNR threshold, the better the performance on the QuickSIN™.

### 2.3. EEG Procedures

#### 2.3.1. Visual Stimuli

All participants were presented with a high-contrast sinusoidal concentric grating that continually transitioned into a radially modulated grating (a circle-star pattern) [2,21,28,39,40,41,42]. This stimulus was presented on a 26-inch flat-screen LCD television at a viewing distance of approximately 42 inches. Each circle and star was shown 150 times, for a total of 300 trials, lasting 600 ms each. Each shape transitioned into the other, giving rise to the perception of apparent motion and shape change to the viewer. The VEP was temporally synchronized to the onset of each individual star and circle image. Participants were instructed to direct their gaze to the center of the star/circle at a black dot, to passively watch the stimulus, and to not shift gaze during the three minutes.

#### 2.3.2. EEG Recording and Analyses

Participants were seated in a comfortable reclining chair in an electromagnetically shielded sound booth and fit with a high-density 128-channel EEG electrode recording net (Electrical Geodesics, Inc, Portland, OR, USA). The visual stimulus was presented via E-Prime^®^ 2.0, (Psychology Software Tools, Inc, Sharpsburg, PA, USA), stimulus software compatible with Net Station 4 (Electrical Geodesic, Inc, Portland, OR, USA).

The sampling rate for the EEG recordings was 1000 Hz, with a band-pass filter set at 0.1–200 Hz via Net Station 4 default settings. Individual continuous EEG data were high-pass filtered offline at 1 Hz using a FIR filter set to Net Station 4 default settings. Low-pass filtering at 30 Hz was only performed for VEP figures created in EEGLAB (see below) using a FIR filter via the function pop_eegfiltnew default settings. Continous EEG data were epoched according to the EEG activity surrounding the stimulus presentation. Each epoch contained a 100 ms pre-stimulus and 495 ms post-stimulus interval. Epoched data were then exported from Net Station and imported in to EEGLAB [43] operating on MatLab^®^ (The MathWorks^®^, Inc, Natick, MA, USA). In EEGLAB, data were downsampled to 250 Hz, implementing a 125 Hz (Nyquist frequency) anti-aliasing filter. This was followed by a pre-stimulus baseline correction and artifact rejection with a criterion of amplitude greater than +/− 100 μV. The data were also analyzed for eye blinks and saccades to remove ocular artifacts. Bad channels were removed from the recording and replaced with interpolated data from the remaining channels via a spline interpolation algorithm. Remaining epochs were averaged and re-referenced using common average reference.

**Figure 2:**
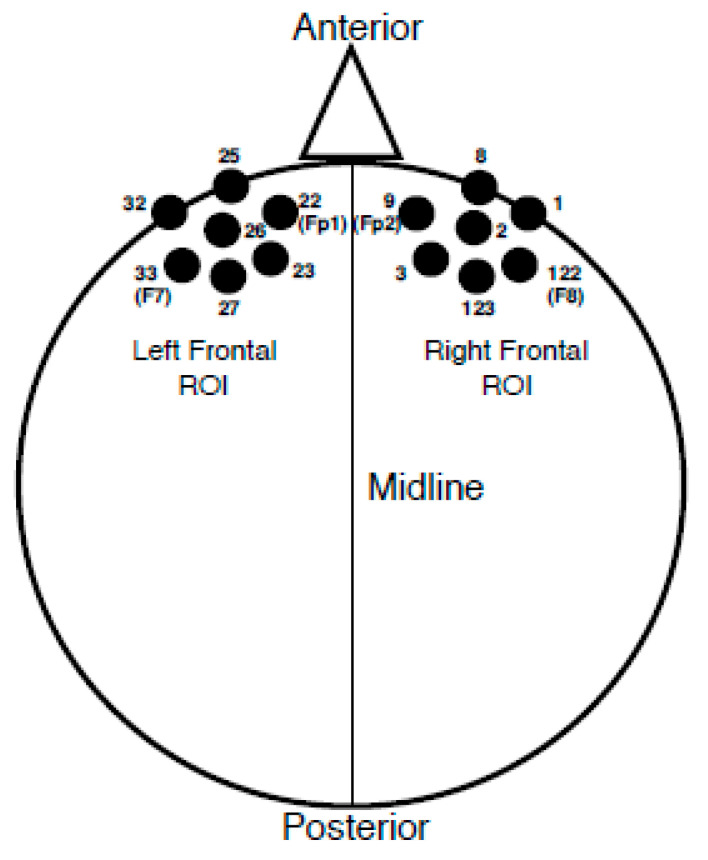
Frontal ROI Electrode Locations. Left frontal ROI electrode locations consist of sensors 22 (10–20 location equivalent of Fp1), 23, 25, 26, 27, 32, and 33 (10–20 location equivalent of F7). Right frontal ROI electrode locations consist of sensors 1, 2, 3, 8, 9 (10–20 location equivalent of Fp2), 122 (10–20 location equivalent of F8), and 123.

Once an individual VEP average was obtained, groupings of seven electrodes corresponding to frontal cortical regions of interest (ROI) were averaged together. Please see Figure 2 for the electrode locations included in each ROI. Electrodes included in each ROI were determined based upon both anatomical location and results from a previous study in which latencies and amplitudes from similar frontal ROIs correlated with speech perception suggestive of cognitive load [35]. VEP peak (P1, N1, P2) amplitude and latency values were recorded in each ROI for each participant. It should be noted that the polarity of the VEP response became negative for the frontal regions as the midline of the scalp was crossed; a result we have observed consistently in our lab by utilizing a common average reference with this particular stimulus [2,40,41], and which has also been observed for VEPs recorded using a common average reference in response to facial stimuli [44]. Therefore, the P1 peak component was designated as the first negative-going peak to occur within a latency window of 90–130 ms, the N1 component as the second peak or first positive-going peak to appear between 150–200 ms, and the P2 component as the third peak or second negative-going peak within 200–300 ms. If a peak component occurred outside of the described latency ranges, it was still marked and included according to the order of appearance (e.g., the first large negative component at 80 ms was marked as P1). Latency and peak amplitudes were recorded at the height of the peak component or at the midway point if the peak was broad. Finally, individual waveform averages were combined and grand-averaged according to MILD and MOD group classifications at each ROI.

### 2.4. Current Density Reconstruction

Independent component analysis (ICA) was performed for individual concatenated EEG data in EEGLAB following artifact rejection and common average referencing prior to ROI averaging [2,35,40,41,45,46,47]. ICA is a statistical method utilized to separate spatially fixed and temporally independent components that underlie the evoked potential [48], important in modeling cortical EEG sources [2,28,35,40,41,46,47,48,49,50]. Once underlying independent components that accounted for the greatest percent variance of the VEP peaks (e.g., P1, N1, and P2) were identified and retained, the ‘pruned’ data (including all 128 channels) were exported into CURRY^®^ Scan 7 Neuroimaging Suite (Compumedics Neuroscan™) for source modeling.

In CURRY, the components were averaged according to each VEP peak and categorized into the MILD and MOD hearing loss groups. For example, each group was comprised of a P1 component average, an N1 component average, and a P2 component average. Separate current density reconstructions (CDRs) were then created for the three VEP component averages in each group, using sLORETA (standardized low-resolution brain electromagnetic tomography). sLORETA is a statistical tool used in estimating CDRs that includes variance of cortical sources in combination with variance from the EEG recording [51,52]. Head models were created using the standardized boundary element method (BEM) geometry [53] in CURRY. Resulting group CDRs were represented by a graded color scale image placed on a Montreal Neurological Institute (MNI) MRI provided in CURRY. Sagittal MRI slices were selected to illustrate the greatest differences in cortical activation between the groups.

### 2.5. Statistical Analyses

Due to the unequal sample size between groups, non-parametric statistical analyses were applied to the data. Peak latency and amplitude were compared separately across groups in each ROI using the Mann–Whitney U test [37]. A one-tailed Spearman’s rank-order correlation was calculated to observe possible relationships between auditory behavioral measures (QuickSIN™ thresholds and high-frequency PTA) and VEP peak component latency and amplitude values in each frontal ROI. All multiple comparisons were corrected for using the Benjamini–Hochberg procedure with a false discovery rate of 0.1 [54].

## 3. Results

### 3.1. Current Density Reconstructions

**Figure 3:**
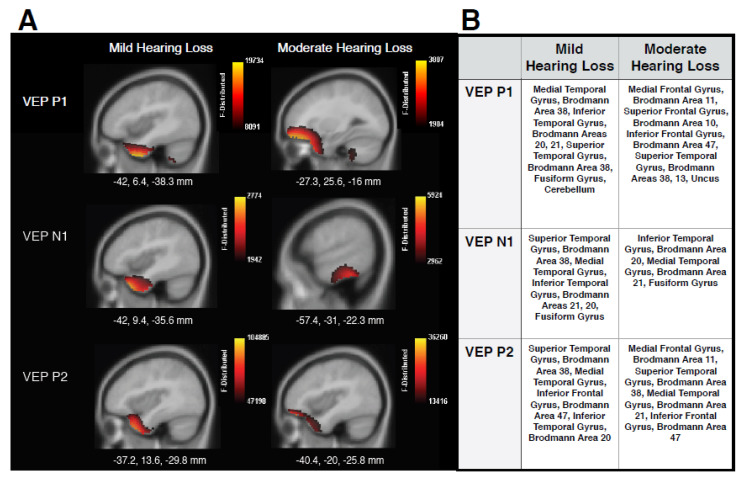
Current Density Reconstructions (CDR)**.** (**A**) CDR images illustrating cortical activation underlying VEP peak components P1, N1, and P1 on sagittal MRI slices for MILD (*n* = 10) and MOD (*n* = 7) hearing loss groups. The scale of the F distribution is shown in the upper right corner ranging from red (lowest level of activation) to yellow (highest level of activation), and Montreal Neurological (MNI) coordinates are listed below the corresponding MRI slice. (**B**) A table listing, in approximate order of highest level of activation, anatomical cortical sources of corresponding VEP components.

Figure 3 shows the CDR images generated using the VEP peak components for MILD and MOD groups in panel A, as well tables listing locations of cortical responses in panel B. The MILD group showed mainly ventral visual cortical activations underlying all VEP components, including superior temporal gyrus (STG), medial temporal gyrus (MTG), and inferior temporal gyrus (ITG). These findings contrast with cerebello-occipital visual activation observed in normal-hearing individuals elicited by the same stimulus [2,17,28,40,41]. Instead, these results are consistent with studies that have reported visual cross-modal re-organization in adults with acquired age-related hearing loss [2,28,29], adults with pre-lingual deafness [7,8,9,13,24,55], and cochlear-implanted adults [16,19,20,21,22,23,26], and supports the hypothesis that sensory deprivation, even to a mild degree, drives visual cross-modal re-organization in a bottom-up manner. Furthermore, sources underlying the later P2 peak began to show minimal involvement of frontal cortical regions, such as inferior frontal gyrus (IFG) and Brodmann Area 47, areas which have also been reported to be responsive to both visual [17] and auditory stimuli in adults with mild-moderate hearing loss [35], and suggestive of preliminary top-down cognitive involvement in cross-modal re-organization. In the MOD group, similar visual re-organization was observed, with contribution from STG to P1 and P2 VEP components, and ITG and MTG involved in the generation of the N1 response. In addition, increased cortical activation between temporal and frontal regions is clearly evident for the MOD group in comparison to the MILD group, and is observed mainly for the P1 and P2 VEP components. It should be noted that we did not find strong activation of frontal cortex underlying the N1 VEP component, as observed by Glick and Sharma (2020). This finding may indicate that cross-modal mechanisms underlying the N1 are not always moderated by top-down resources, or that frontal networks become increasingly active across components with a greater degree of hearing loss that was not present in this study. In any case, the involvement of frontal sources for the P1 and P2 components is consistent with studies reporting frontal activation in response to visual and auditory input for adults with mild-moderate hearing loss [28,31,35] and supports the hypothesis that increasing visual recruitment of cognitive networks occurs as hearing loss severity increases. Thus, top-down mechanisms may modulate cross-modal visual processing according to hearing loss severity.

### 3.2. Visual Evoked Potentials

**Figure 4:**
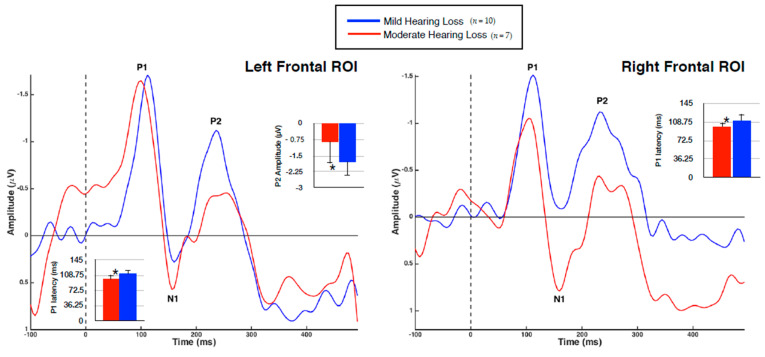
Group Averaged VEPs of Frontal Cortical ROIs. Average VEP waveforms in the left and right frontal ROIs for the MILD hearing loss group (*n* = 10) are represented in blue, while the MOD hearing loss group (*n* = 7) is represented in red. VEP waveforms are shown as amplitude functions (vertical axis in microvolts) over time (horizontal axis in milliseconds). Bar graphs illustrate significant differences, with one asterisk denoting significance at *p* < 0.05.

VEP group averages according to ROI are shown in Figure 4, with significant mean differences illustrated via bar graphs. Note that the polarity of the VEP response becomes negative for the frontal regions as the midline of the scalp is crossed, consistent with similar studies [2,40,41,44]. As expected, each group demonstrated three obligatory VEP peaks (P1, N1, P2) in all regions [44].

It was found that the MOD hearing loss group demonstrated an earlier VEP P1 latency than the MILD group in both the left (*U* = 12, *Z* = −2.27, *p* < 0.05) and right (*U* = 11, *Z* = −2.39, *p* < 0.05) frontal regions. The finding of decreased VEP latency outside of primarily visual processing regions is suggestive of cross-modal recruitment as a result of hearing loss [2,26,28,40,41], and may suggest a more efficient visual processing network. Amplitude differences between the groups were also identified, with the MOD hearing loss group showing reduced P2 amplitude in the left frontal cortex (*U* = 59, *Z* = 2.34, *p* < 0.05). Although increased VEP amplitude is typically associated with cross-modal recruitment and indicative of strengthened neural networks [2,14,15,21,22,40], reduction of VEP amplitude has been reported after training in visual tasks [56,57], again indicative of a more efficient visual processing network. Finally, it should be noted that while these findings were statistically significant, the small sample size in each group should be considered and results interpreted with caution.

### 3.3. Speech Perception in Noise and VEPs

**Figure 5:**
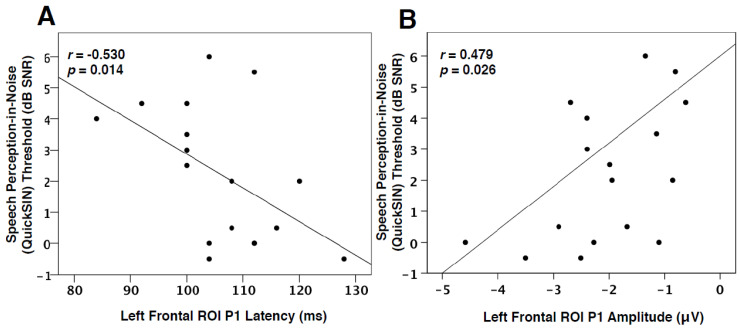
Quick SIN ^TM^ and VEP Frontal ROI Correlations. (**A**) The correlation of VEP P1 latency in the left frontal ROI as a function of QuickSIN™ threshold values. P1 latency is shown on the horizontal axis in milliseconds and threshold values on the vertical axis as signal-to-noise ratio in dB (dB SNR). Note that a lower threshold indicates good auditory performance. The Spearman’s rank order correlation value and significance level are shown in the upper left corner. (**B**) The correlation of VEP P1 amplitude in the left frontal ROI as a function of QuickSIN™ threshold values. P1 amplitude is shown on the horizontal axis in microvolts.

Due to the CDR results showing frontal generators for VEP P1 and P2 components (Figure 3), as well as between-group differences observed for these peaks (Figure 4), a one-tailed Spearman’s rank-order correlation was performed to observe possible relationships between the latency and amplitude of these components and QuickSIN™ threshold values for both groups. Although no significant difference was found between the QuickSIN™ threshold values for the MILD and MOD groups (*U* = 48.5, *Z* = 1.32, *p* > 0.05), significant correlations were identified between SNR thresholds and VEP components. As seen in Figure 5A, speech perception in noise thresholds was negatively correlated with VEP P1 latency in the left frontal ROI (*r* = −0.530, *p* = 0.014, Figure 5A). Thus, it appears that greater difficulty in speech perception is related to concurrent decreases in VEP P1 latency in the left frontal cortex, suggestive of a frontal network that supplements visual processing as speech perception becomes more effortful. Similar relationships in adults with mild-moderate hearing loss have been reported between decreases in VEP latency and poor speech perception in temporal ROIs, indicative of cross-modal re-organization [2,28]. Along these lines, P1 amplitude in the left frontal ROI positively correlated with speech perception in noise, such that a decrease in amplitude (or amplitude becoming more positive-going) coincided with worse speech perception (Figure 5B). However, as this finding became non-significant following a correction for multiple comparisons, we discuss it as a trend. Decreases in VEP amplitude may occur following task-specific training [56,57] suggestive of greater synaptic efficiency. Taken together, the association between decreased VEP latency and the trend for reduced amplitude in frontal regions with more effortful speech perception illustrates top-down plasticity and modulation of visual processing as speech perception ability decreases and listening effort increases. This interpretation is consistent with findings that demonstrated associations between increased listening effort and visual activation of frontal networks in hearing loss [31] and agrees with our hypothesis that top-down involvement becomes increased as auditory performance decreases.

### 3.4. Hearing Loss and VEP Amplitude

**Figure 6:**
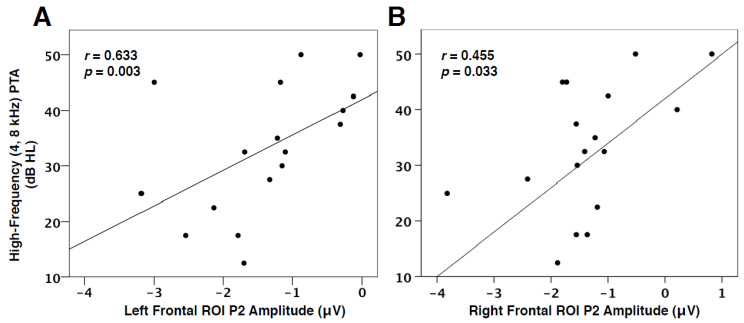
PTA and VEP Frontal ROI Correlations. (**A**) The correlation of VEP P2 amplitude in the left frontal ROI as a function of pure-tone threshold averages (PTA) at 4000 and 8000 Hz (best ear). P2 amplitude is shown on the horizontal axis in microvolts and threshold values on the vertical axis as decibels hearing level (dB HL). The Spearman’s rank order correlation value and significance level are shown in the upper left corner. (**B**) The correlation of VEP P2 amplitude in the right frontal ROI as a function of high-frequency PTA.

In addition to the link between decreased speech perception and VEP characteristics, we found the degree of hearing loss to be positively correlated with VEP P2 amplitude in the left (*r* = 0.633, *p* = 0.003) and right frontal cortices (*r* = 0.455, *p* = 0.033), as seen in Figure 6. In other words, as hearing loss increases, the amplitude of the visual response, as represented by the P2 component, in the frontal cortex decreases (becomes more positive-going). This finding is consistent with VEP amplitude reduction, suggestive of more efficient processing, observed post-training in visual tasks [56,57], and reports of early-stage auditory deprivation intiating frontal cortical plasticity in visual processing [28,31]. This result therefore supports the hypothesis that cognitive networks may be more relied upon and facilitate cross-modal processes as hearing loss worsens.

## 4. Discussion

In this study, our aim was to assess whether frontal cortical networks may facilitate visual processing and re-organization in a top-down manner as hearing loss progresses in severity from mild to moderate. To achieve this goal, we recorded VEPs in response to apparent motion using high-density EEG in adults with mild high-frequency hearing loss and adults with moderate high-frequency hearing loss. We examined visual cortical generators between adults with mild high-frequency hearing loss and adults with moderate high-frequency hearing loss using CDRs generated via sLORETA. In addition, we correlated auditory behavioral performance (i.e., speech perception in background noise and high-frequency hearing thresholds) with VEP component characteristics to evaluate possible relationships between auditory behavioral performance and visual plasticity.

Our results are comprised of three main findings: (a) increased recruitment of frontal cortices combined with cross-modal recruitment of temporal auditory regions for visual processing in moderate hearing loss, (b) significantly decreased VEP latency and amplitude in frontal cortices for moderate hearing loss, and (c) significant correlations between VEP characteristics and auditory behavioral measures. These results are all suggestive of top-down mechanisms which likely modulate cross-modal visual processing, at least to some degree, according to hearing loss severity.

### 4.1. Frontal Top-Down Modulation in Hearing Loss

Our previous findings have consistently shown that the visual stimuli described in the present study activate cerebello-occipital visual networks in individuals with normal hearing [2,17,28,40,41]. In contrast, the results of the current study show that visual activation in adults with mild hearing loss includes auditory temporal regions, accompanied by a gradual increase in frontal processing underlying the later visual cortical response (Figure 3). Thus, the MILD group demonstrates evidence of visual cross-modal re-organization, likley driven by bottom-up sensory deprivation of a mild degree. As hearing loss progresses, represented by the MOD group, visual processing increasingly draws upon frontal networks, concurrent with visual recruitment of auditory regions. These data illustrate both visual cross-modal re-organization and a growing involvement of top-down modulation typically associated with cognitive processing of sensory information [31] with increased hearing loss severity. It should be noted that the participants in this study were passively observing visual stimuli, or were not required to perform a task during viewing [2,17,28,40,41]. Therefore, as hearing loss increases, it appears that frontal networks modulate low-level stages of cross-modal visual processing in temporal regions, possibly ‘priming’ resources such as attention for engagement [18]. Such findings are consistent with passive listening of auditory input for listeners with similar degrees of hearing loss [35], and suggest increased cognitive load for sensory processing.

Previous studies have identified specific frontal networks to be related to listening effort and hearing loss severity during auditory and visual tasks. For example, the left IFG, a region that was active during the VEP P2 component in the MILD group and across all VEP components in the MOD group, appears to act as a supplementary network for speech perception recovery in cochlear-implanted adults [23] and has been shown to be involved in degraded auditory and working memory tasks [58,59]. The left IFG also presents with an increased response in adults with mild-moderate hearing loss in response to auditory, visual, and audio-visual speech perception tasks, which is correlated with hearing loss severity [31]. Similarly, Brodmann Areas 10 and 11, identified as sources of VEP components in the MOD group, are cortical regions indicated to support information encoding, learning, and anticipation of/attention to incoming sensory input [60,61,62,63], and are increasingly active during speech perception in auditory and visual tasks for listeners with mild-moderate hearing loss [31]. Finally, functional connectivity studies have demonstrated increased top-down connectivity between inferior frontal and auditory cortices during speech perception tasks for adults with hearing loss, while listeners with normal hearing demonstrate typical bottom-up connectivity [64,65]. The results of the current study show that visual activation in adults with mild hearing loss includes auditory temporal regions accompanied by a possible increase in connectivity with frontal processing. This connectivity is represented by a continuous source current between frontal and temporal regions (Figure 3) for the MOD group in the P1 and P2 VEP components. Future studies should directly examine connectivity changes which underlie cross-modal enhancement and cognitive upregulation in persons with hearing loss. Overall, our findings are in line with previous research and suggest that cognitive-related areas in the frontal cortex aid in cross-modal processing of visual information in temporal cortices as hearing loss increases in severity, with the likely end goal of optimizing compensatory audio-visual function [14,15,31].

While top-down modulation of auditory, visual, and audio-visual networks is indicated to supplement auditory cognitive function [28,31,66,67], recruitment of these networks, especially at pre-cognitive stages, may place added demands on such resources and increase cognitive load. For instance, we have found that passive listening of auditory stimuli mainly elicits a response in the frontal cortex for adults with mild-moderate hearing loss, while adults with normal hearing demonstrate expected temporal auditory sources [35]. This early and consistent involvement of executive systems in processing degraded auditory input may contribute to the link between hearing loss and cognitive decline in older adults with and without dementia [5,67,68,69,70] as cognitive networks that are normally reserved for higher-order tasks are always, essentially, ‘on’. In this study, we show an additional potential draw upon cognitive resources for the processing of low-level visual information in temporal regions as hearing loss increases.

### 4.2. Evoked Potential Indices of Cortical Plasticity in Hearing Loss

Evoked potential latency has been consistently reported as a biomarker of both visual and auditory cortical plasticity in hearing loss [17]. For example, decreased VEP latency recorded in temporal regions is reflective of visual cross-modal reorganization and corresponds with poor outcomes in auditory performance for adults with hearing loss [2,28]. In the current study, we observed decreased VEP P1 latency in bilateral frontal cortices for the MOD group, which to our knowledge has not been described previously in the hearing loss literature. This finding suggests an early occipital-frontal network that is processing visual input more efficiently in moderate versus mild hearing loss. On the other hand, individuals with hearing loss have also shown increases in latency of cortical auditory evoked potentials (AEPs) in frontal and central cortices, which have been correlated with deficits in auditory cognitive performance [35,71]. Therefore, while visual networks decrease in processing time, there appears to be a coinciding increase in auditory processing time for adults with mild-moderate hearing loss [35,71]. This indicates that a tradeoff between the efficiency of frontal processing of auditory and visual information could be initiated in hearing loss. Due to a gradual decrease of bottom-up auditory input, the frontal cortex may modulate visual and auditory function simultaneously for compensatory purposes. This modulation could result in the strengthening of non-deprived visual networks while increasing resources to interpret incoming auditory information that is degraded (i.e., ‘effortful’ listening) [17,66].

Evoked potential amplitude changes have also been taken as evidence of cortical plasticity. For example, increased VEP amplitude in auditory cortical regions occurs in visual cross-modal re-organization and is correlated with decreased speech perception [16,19,21,22], but also increased audio-visual integration in hearing loss [14,15]. However, the present study found a significant decrease in VEP P2 amplitude (toward the positive-going direction) in the left frontal cortex (Figure 4) in the MOD group. One possible explanation for this discrepancy is that training effects in the visual modality have been shown to result in decreased VEP amplitude in the frontal cortex [56,57], which may represent an increase in network efficiency (similar to decreased VEP latency). Along these lines, increased amplitude of auditory evoked potentials in frontal and central cortices related to mild-moderate hearing loss [35,71] could signify ‘effortful’ listening [72], or a decrease in network efficiency. Therefore, just as in the tradeoff found between VEP and AEP latencies in the frontal cortex, it appears that a similar relationship in functional efficiency is also represented by decreased VEP amplitude and increased AEP amplitude in the frontal cortex. In addition, we recommend that these group comparison findings be interpreted with caution, due to the small sample size in each group. Future studies should aim to extend the participant pool when performing similar analyses.

### 4.3. Visual Cortical Plasticity and Auditory Behavioral Outcomes

Visual cross-modal reorganization of auditory cortices has been previously illustrated to correlate with poor speech perception performance and cognitive performance [2,22,23,28]. This is likely due to a competition for resources taking place as visual networks recruit auditory resources and lower-level auditory processing moves to frontal systems [2,17,35]. In order to determine whether top-down modulation of visual function may be associated with speech perception performance, we correlated individual speech perception in noise thresholds with VEP P1 and P2 latency and amplitude values in left and right frontal ROIs. As shown in Figure 5A, VEP P1 latency in the left frontal ROI was found to negatively correlate with speech perception in noise thresholds. In other words, left frontal activation becomes faster in response to visual information, and speech perception thresholds appear worse. Similarly, there was a trend for decreased P1 amplitude in the left frontal cortex to correlate with decreased speech perception (Figure 5B). Again, these results illustrate an increasingly strengthened top-down network that becomes active early in visual processing for adults with worsening speech perception. Although inconsistent with research describing increased visual function in the frontal cortex to coincide with improved speech perception in cochlear-implanted adults [15,23], this result has also been found in studies reporting increased cognitive load to lead to decreased auditory performance [35,66,67,73,74]. Thus, cognitive resources may be taxed by either auditory or visual modalities for compensatory purposes in hearing loss. Finally, a positive relationship between VEP P2 amplitude in left and right frontal cortex and hearing loss severity (Figure 6) was identified, signifying that increasing hearing loss (and consequently a longer duration of auditory deprivation) is associated with a possible increase in cross-modal network efficiency in frontal cortices.

While bottom-up visual cross-modal re-organization and top-down modulation of visual function is indicated to correlate with poor auditory behavioral outcomes, this plasticity may be beneficial to audio-visual integration in hearing loss in real life situations [14,15,29,31], illustrating the compensatory role of visual re-organization. However, if visual compensation no longer serves a compensatory function, cortical organization may revert back to typical function as observed in adults with normal hearing, and evidenced by recent research. Glick and Sharma (2020) investigated amplification effects on visual cross-modal re-organization in adults with mild-moderate hearing loss, and found that, post-6 months intervention, adults with hearing aids no longer demonstrated evidence of visual cross-modal re-organization and frontal activation during visual stimulation. Furthermore, these changes were associated with individual functional gains in speech perception and cognitive measurements. In contrast, studies in deaf adults and children with cochlear implants show evidence of visual cross-modal recruitment and frontal activation even after years of CI use [21,23,40]. These findings suggest that, at least in early-stage hearing loss, cross-modal plasticity associated with decreased cognitive outcomes is not permanent, and may be reversed if addressed in time.

## 5. Summary and Conclusion

Our results present evidence of early top-down modulation of visual processing in temporal areas as hearing loss severity progresses from mild to moderate degrees. At the same time, visual cross-modal reorganization of auditory regions is observed to be stable in both mild and moderate hearing loss. This frontal modulatory network, which includes areas of executive function, is negatively related to speech perception in background noise. Taken together, it is apparent that top-down modulation of visual function increases according to the degree of hearing loss, following bottom-up-driven visual recruitment of auditory systems, for compensatory purposes. At the same time, this increasing draw upon cognitive resources may co-occur with re-allocation of auditory function to frontal cortices for low-level sensory processing in hearing loss. It is therefore possible that these compensatory mechanisms may contribute to cognitive decline as finite resources continue to be taxed over time. Future studies should address the relationship of frontal and cross-modal compensation with cognitive performance in persons with hearing loss.
